# Hand Transplants, Daily Functioning, and the Human Capacity for Limb Regeneration

**DOI:** 10.3389/fcell.2022.812124

**Published:** 2022-03-04

**Authors:** Susan M. Fitzpatrick, David Brogan, Prateek Grover

**Affiliations:** ^1^ James S. McDonnell Foundation, St. Louis, MO, United States; ^2^ Department of Orthopaedic Surgery, Washington University in St. Louis School of Medicine, St. Louis, MO, United States; ^3^ Division of Neurorehabilitation, Orthopaedic Surgery and Neurology, Washington University in St. Louis School of Medicine, St. Louis, MO, United States; ^4^ The Rehabilitation Institute of St Louis, St. Louis, MO, United States

**Keywords:** regeneration, transplantation, microsurgery, functional, hand, prosthesis and implants, rehabilitation, delivery of care

## Abstract

Unlike some of our invertebrate and vertebrate cousins with the capacity to regenerate limbs after traumatic loss, humans do not have the ability to regrow arms or legs lost to injury or disease. For the millions of people worldwide who have lost a limb after birth, the primary route to regaining function and minimizing future complications is *via* rehabilitation, prosthetic devices, assistive aids, health system robustness, and social safety net structures. The majority of limbs lost are lower limbs (legs), with diabetes and vascular disorders being significant causal contributors. Upper limbs (arms) are lost primarily because of trauma; digits and hands are the most common levels of loss. Even if much of the arm remains intact, upper limb amputation significantly impacts function, largely due to the loss of the hand. Human hands are marvels of evolution and permit a dexterity that enables a wide variety of function not readily replaced by devices. It is not surprising, therefore, for some individuals, dissatisfaction with available prosthetic options coupled with remarkable advances in hand surgery techniques is resulting in patients undertaking the rigors of a hand transplantation. While not “regeneration” in the sense of the enviable ability with which Axolotls can replace a lost limb, hand transplants do require significant regeneration of tissues and nerves. Regaining sophisticated hand functions also depends on “reconnecting” the donated hand with the areas of the human brain responsible for the sensory and motor processing required for complex actions. Human hand transplants are not without controversy and raise interesting challenges regarding the human regenerative capacity and the status of transplants for enabling function. More investigation is needed to address medical and ethical questions prior to expansion of hand transplants to a wider patient population.

## Introduction

As much of the biological research and medical community continues to associate limb regeneration with invertebrates or a few selected vertebrate examples, the limits of regenerative capacity in adult humans, particularly for limbs, retain its influence on research and care. A recent Lancet Commission report provides an overview of the challenges to mainstreaming regenerative medicine (Cossu et al., 2018). In this perspective, we evaluate the extent to which human hand transplantation serves as a major exemplar reflecting the human capacity for limb “regeneration” across biological scales, from cells and organ systems to restore everyday function. The scope of the perspective is focused on transplantation and function. We do not discuss the ramifications of congenital loss of limb in humans, nor do we attempt to provide a systematic review of the medical and surgical management of hand replantation or transplantation; several recent reviews that do so are available (Foroohar et al., 2011; Errico et al., 2012). Rather, we offer our perspective that the attachment of a cadaver donor hand to an individual who has lost a hand can reveal the capacities of *adult* human limb regeneration (up to 8 weeks of age, human embryos can regenerate a limb), including tissue regeneration and functional recovery, enabled by appropriate postsurgical rehabilitation programs. We are not asserting that attaching a donor hand to an amputee’s forearm is the *same as* growing a new hand. However, the very premise of integration of a donor hand within an individual’s physical form manifests the ability of skin, muscles, tendons, blood vessels and nerves to undergo substantial regeneration, repair and remodeling.

To be ultimately successful from the patients’ perspective, hand transplants must achieve remarkable feats of functional recovery. The specialized function of the human hand with respect to dexterity, grasp, and completion of complex actions, requires coordination across brain regions ranging from primary motor and sensory cortices to integrative regions such as the premotor/parietal areas (Corbetta and Fitzpatrick, 2011; Frey et al., 2011). The restoration of hand function (such as reaching, grasping or pinching) after severe trauma requires rehabilitation strategies focusing not only on primary motor and sensory cortices, but also on recruiting cortical brain areas related to motor planning and action (Pomeroy et al., 2011; Frey, 2015). Much of what we know about rehabilitation of upper limb action has been learned from patients and animal models with brain lesions and peripheral nerve injuries. Effective translation of the body of knowledge focused on central lesions and peripheral nerve trauma into optimal therapy protocols for individuals with hand transplants will require substantially more research. Current efforts in this area are hampered by limited case study reports available from what is considered an experimental therapy.

Following the loss of a hand, skills that would have reached a high level of proficiency and automaticity in adults need to be relearned and often accomplished with various strategies, including use of a prosthetic device. There is a robust literature on the impact of peripheral damage as manifested in the functional organization of primary motor and sensory cortex and brain areas related to complex actions, but mechanistic understanding remains incomplete (Makin et al., 2015; Makin and Flor, 2020). Because relatively few human hand transplants are performed each year, less is known regarding the cortical changes accompanying hand loss followed (sometimes years later) by hand transplantation (for case study see Madden et al., 2019). For example, with peripheral nerve regeneration proceeding at a rate of 1 mm/day after nerve transection (Fu and Gordon, 1997), the sensory input from the donor hand to the brain will be degraded in comparison with that from an intact hand during recovery from transplantation surgery. Yet case studies indicate that some aspects of hand function return quicker than would be anticipated (Neugroschl et al., 2005; Frey, 2021). There are other instances where the brain has the capacity to functionally adapt and relearn from the availability of even impoverished stimuli, such as the ability of individuals with cochlear implants to recognize and interpret vocal speech (Peterson et al., 2010).

## The Ramifications of Hand Loss

The loss of a hand through injury and amputation can impact both avocation and vocational activities. Temporary loss of hand use (for example when bandaged) quickly causes even the simplest and most routine of everyday tasks to become frustratingly clumsy and inefficient. Hands also play central roles in our social and cultural lives and the symbolism of hands as central to our humanity can be found in works of art, music and literature (Wilson, 1998). Aristotle’s observation that the human hand is the “tool of tools” pays homage to its functionality. Recent studies have demonstrated that our fingertips can detect differences in surfaces altered at the molecular level (Nolin et al., 2021). Reaching out to grasp a coffee cup, manipulating the button of a shirt, or pinching a minute quantity of salt while cooking are difficult tasks to replicate with present day robotic systems. Moreover, our hands are tightly coupled to our sense of self and the expressions of our personal identity. The uniqueness of our fingerprints, the individuality of our signatures, even our choice of clothing reflects the abilities of our hands. Individuals who have lost a hand to amputation are bothered by the compromises they make—for example wearing “pull on” clothes to dress independently (Frey, 2021). Our hands physically connect us with the world and with family and friends. When meeting complete strangers, it is not uncommon in many cultures that first greetings involve some actions of our hands.

Hand transplantation is not without controversy, particularly because of the need for life-shortening immunosuppression for a non-life-sparing intervention (see further discussion below). However, the loss of a hand from trauma or amputation is life-altering, with some patients experiencing deep dissatisfaction with prosthetic devices. In such cases, the desire for transplantation can be worth the risk and the effort (Frey, 2021). Recovery of hand function is dependent on the reparative regeneration of skin, tendons, muscle, vasculature, and peripheral nerves, and demonstrates that adult humans have significant regenerative capacity at the tissue level. While skeletal muscle, bone and nerve regeneration is necessary, it alone is not sufficient for skilled use of a donor hand.

It is also necessary that cognitive control accurately direct the actions of the donor hand in a fashion like that of the native hand, highlighting the essential role of cortical regeneration. Early concerns regarding the limited capacity for functional recovery due to reorganization of cortical sensory and motor areas were informed by studies with mature primate brains following injuries or amputations (for review see Gunduz et al., 2020 and Andoh et al., 2020 and references within). Studies in both humans and animals have found that areas of the brain dedicated to the neural representation of the hand respond to sensory stimulation of the face after upper limb loss (Ramachandran and Rogers-Ramachandran, 2000). Additionally, recent work indicates that there is a capacity, post amputation, for the brain to retain neural representations of missing limbs (Kikkert et al., 2016), Functional imaging studies of hand transplant patients support our current understanding that there is indeed “regeneration” of neural representations for action in the sensory and motor control areas in the central nervous system (Valyear et al., 2019), even when transplantation occurs many years after the loss of a hand.

## Hand Loss in the Context of Limb Loss and Prosthetic Limb Use

For acquired upper limb loss, trauma is the primary etiology, with digit loss the most common amputation level. Atroshi and Rosberg, (2001) While sources such as the National Limb Loss Resources Center [National Limb Loss Resource Center® - Amputee Coalition (amputee-coalition.org)] and National Trauma Databank [ntdb rds user manual all years.ashx (facs.org)] report limb loss statistics, the incidence and prevalence of upper limb loss is not as well characterized as lower limb loss. Best estimates placed the prevalence in the United States in 2005 close to half a million people, with approximately 90% categorized as minor or digital only (Ziegler-Graham et al., 2008) and millions more worldwide. A more recent estimation by the National Trauma Databank using 2009–2012 data places the prevalence at 46 per 100,000 NTDB trauma admissions (Inkellis et al., 2018). The global burden disease data tool (GBD Results Tool | GHDx (healthdata.org) provides global incidence, prevalence and years lived with disability (YLD) data. From 1990 to 2019, for unilateral upper limb amputation, global incidence has increased from 38 to 67 thousand, prevalence from 1.16 to 2.1 million, and YLD from 75 to 115 thousand. However, the numbers, startling as they are, cannot adequately capture the impact on quality of life for an individual experiencing limb loss.

An internationally accepted framework with the potential to enrich our knowledge of the functional consequences of limb loss is the World Health Organization International Classification of Functioning, Disability and Health (WHO ICF) (https://www.who.int/standards/classifications/international-classification-of-functioning-disability-and-health). Some studies in the orthotics and prosthetic device literature have tried to use this framework in the clinical setting to systematically monitor function and barriers to use (Burger 2011), but this approach has not yet found widespread application. However, the WHO ICF remains one of the frameworks to bring together all stakeholders in the multidisciplinary field of limb loss for clinical and policy impact. Using the WHO ICF, upper limb loss entails a change in anatomic and physiologic function (*impairment*) that has daily activity (*activity*) and work, recreation, personal and driving related implications (*participation*). As in many cases of debilitating injuries the contextual factors including the *environment* and *inter- and intra-personal factor*s contribute to the variable nature of individual-level outcomes.

The journey of a person with upper limb loss back to community participation is long, requiring a robust system of care that enables risk factor modification, timely rehabilitation, and prosthetic device provision (Pasquina et al., 2015). Upper limb prosthetic device options exist to meet a range of functional needs ranging from heavy physical labor to fine motor skills. In general, mechanical devices are more suited to the former and newer electronic/hybrid devices better suited to the latter (Carey et al., 2015). Several technological advances have been developed over the last half century to improve prosthesis function, including targeted muscle reinnervation (Kuiken et al., 2009) and osseointegration (Diaz Balzani et al., 2020). Nevertheless, the rate of abandonment of devices is reported to range from 9 (Yamamoto et al., 2019) to 20% (Biddiss and Chau, 2007), with anecdotal evidence placing this number closer to 50% or more. This begs the question, would publication of higher rates of device abandonment serve as ammunition for denial of prosthetic devices even for appropriate prosthetic device candidates, or would it promote development of better devices and treatment alternatives such as transplantation? Another perspective to consider is the actual definition of abandonment itself, especially across disciplines. Some amputees may use their devices infrequently, for specific situations only, or may stop using the prosthesis during a period of illness, only to resume use once they are better. Hence, time-bound and situation-specific criteria need to be built into the definition of prosthetic device abandonment by limb care professionals in discussion with device users. Health equity must also be considered: Comprehensive systems such as the Veteran’s Administration Amputation System of Care enable different access than non-Veterans Administration beneficiaries and hence, the impact of abandonment is likely felt differently across different patient populations in different systems.

Factors cited for abandonment relate to limb loss (level and etiology), sociodemographics (gender), the prosthetic device (comfort, perceived utility) and system of care (time to fitting, patient enablement for component selection) (Biddiss and Chau, 2007). Given that cosmesis and utility are recurring themes (Ritchie et al., 2011; Yamamoto et al., 2019) for abandonment, a natural question is whether upper limb transplantation offers alternatives for successfully addressing limb loss related impairments, activity limitations and participation restriction.

## Why are Hand Transplantations so Rare?

The technical considerations of hand transplantation can appear daunting, but their success is rooted in the collective experience of limb replantation. The era of modern microsurgery in the United States was heralded by successful replantation of a young boy’s arm by a team of 12 surgeons in a two-stage procedure in 1962 (Malt and McKhann, 1964). Since those early days, advances in the field of microsurgery have enabled ever more sophisticated reconstructive options, through the iterative development of refined microsurgical equipment. Development of improved vascular clamps, microsurgical forceps and intra-operative microscopes facilitated more precise technical work, in parallel with refinements in nerve repair (Tamai, 2009). The growing interest in microsurgery as a field was accompanied by the first reported hand transplant in 1964 in Ecuador^1^ (Fernandez et al., 2019). This early attempt was complicated by acute rejection resulting in amputation within 3 weeks. Subsequent improvement in immune modulation techniques led to the second and third hand transplants performed in 1998 and 1999, with long term graft survival (Foroohar, et al., 2011). Further advances in immunosuppression combined with enhanced microsurgical technique and osseous fusion techniques have enabled the establishment of multiple hand transplant centers throughout the world (Lee, 2017). However, as discussed below, ethical concerns about relative risk versus benefit prevent its widespread application to all upper extremity amputees.

Solid organ transplant is well accepted as a satisfactory technique to prolong life with clinically acceptable risks (Linden, 2009); thus, it is tempting to assume that the ethical concerns of using allograft tissue would have been put to rest. Yet, hand transplants (and now face transplants) are unique among composite tissue allografts in that they do not prolong life, but instead improve function. In fact, receipt of a hand transplant and use of the prescribed immunosuppression regimen may actually shorten the recipient’s life secondary to development of chronic medical conditions such as cytomegalovirus infection, diabetes (Ravindra et al., 2008) or cardiovascular disease (Boratyńska et al., 2014). In recognition of the need for guidance to weigh the health risks of immune suppression with the potential benefit of the transplanted hand, several decision analysis studies have been performed (Chung et al., 2010; Alolabi et al., 2015). In these models, there exists an increasing recognition that the ultimate function of the hand (and not just survival) will influence the decision analysis, particularly for unilateral hand amputees (McClelland et al., 2016). In comparing risks and benefits, the years of life lost or medical comorbidities gained because of immune suppression are weighed against the relative increase in function compared to the base case of prosthesis use. Refinement of immune modulation techniques may in the future tip the decision tree further to hand transplantation, but this will likely be countered in some part by advances in prosthetic limb function.

Dozens of papers concerning the ethics of hand transplantation have been published over the past two decades, mostly focusing on non-maleficence (importance of doing no harm) as well as patient autonomy (recognizing the need for thorough informed consent (Cooney et al., 2018). Extrapolation to the adult population is in question, it is interesting to note that a pediatric Monte-Carlo simulation found that while compared with prosthetic limbs, bilateral hand transplants offered slightly more quality adjusted life years, while unilateral hand transplants were inferior (Snyder et al., 2019). Notably, this did not account for overall cost, just the utility of the intervention, but a key determinant of the risk benefit ratio was the willingness of the patient and family to accept a potentially shortened life span due to the deleterious effects of the required immune suppression.

## Technical Considerations

Although sharing some technical overlaps, hand transplants differ in many ways from the reattachment, or replant, of an individual’s own hand following trauma. Although it might seem counterintuitive, transplants present ideal conditions for tissue harvest of the non-traumatized allograft, compared to a potential extensive zone of injury in replanted hands. For transplantation, the surgeon harvests the donor hand at a level that matches the intended recipient’s deficit. Harvest through forearm musculature may prove difficult to reconstruct extrinsic flexor and extensor tendon function, therefore optimal reconstruction may involve harvest through the distal third of the forearm (where only tendons are found) or through the elbow, prior to the majority of the motor branches to the forearm musculature. Once the hand is transferred to the recipient, teams of surgeons work to stabilize the bony anatomy with plates and screws, followed by sewing of the extrinsic forearm tendons, and establishment of blood flow with microsurgical repair of the major blood vessels to the forearm and hand. Finally, the radial, ulnar and median nerves are repaired by coapting the cut nerve ends with microsurgery. In the near term, survival of the transplant is dependent on patency of the vascular anastomoses, with particular care given to monitoring for intravascular thrombosis. Optimal function in the medium term is dependent on union of the donor and recipient forearm bones, healing of the tendon transfers with minimal adhesions and ultimate neural regeneration to provide a sensate, functional hand.

## Medical Considerations

In fact, while the techniques to perform hand transplants have been refined through more than five decades of replant experience, long term survival depends in large part on prevention of allograft rejection. However, functional success depends on successful union of bone and tendon between donor and recipient parts and neural regeneration from the recipient into the allograft. In fact, the critical importance of neural regeneration is underscored by the fact that early ethical concerns of hand transplant revolved around the unknown functional result of such a procedure, particularly with regards to success of peripheral nerve regeneration.

Host nerves are coapted to the allograft nerves during the transplant procedure and must grow along the length of the donor nerve scaffold and reinnervate end organs (skin or muscle). Transcriptional and translational changes in the proximal and distal nerve stumps lead to a host of alterations in the molecular environment to help this regeneration across the nerve gap at a typical rate of 1 mm/day (Fu and Gordon, 1997). Interestingly, immune suppression appears to potentiate the regenerative capability of peripheral nerves, with particular benefit seen in the local and systemic administration of tacrolimus (Zuo et al., 2020). Clinical reports have noted increased rates of nerve regeneration, up to 2–3 mm per day in hand transplant patients on immune suppressive regimens and may account for the evidence of early functional recovery Jones et al., 2000).

Hand transplants differ from solid organ transplants as they are composite tissue, consisting of muscle, skin, tendon, nerve and bone. Each of these components pose a unique risk profile for immunogenicity (Murray, 1971), with rejection of the skin component often serving as the first sign of graft compromise due to its highly immunogenic nature (Schneeberger et al., 2013) ^2^ attributed to the presence of resident T-cells (Leonard et al., 2020). Ease of monitoring of skin leads to high rates of success in treatment of acute rejection, despite a prevalence of more than 80% in vascularized composite allografts. Typical immunosuppressive regimens begin with induction therapy (antithymocyte globulin) designed to deplete hosts T-cells, followed by maintenance therapy consisting of steroids and tacrolimus (Kueckelhaus et al., 2016).

## Rehabilitation and System Components

The success of this highly multidisciplinary field depends upon a well-coordinated robust system of care (Amirlak et al.). It is not surprising that hand transplant programs are centered in academic medical centers such as Leeds Teaching Hospitals NHS Trust (Burdon et al., 2020), Brigham and Women’s Hospital (BWH) (Bueno et al., 2014) and University of Kentucky (Amirlak et al., 2007). The multidisciplinary team should include surgeons, transplant specialists, coordinators, mental health professional and rehabilitation professionals including therapists (Ravindra and Gorantla, 2011). The role of Physical Medicine and Rehabilitation (PM&R) is not explicitly defined in current literature but needs to be strongly considered. Post-operative hand therapy is critical to successful restoration of extrinsic hand function while protecting tendon transfers. Numerous published protocols exist to direct hand therapists in the care of flexor (Starr et al., 2013) and extensor tendon repairs (Collocott et al., 2018) alongside dedicated hand transplant rehabilitation protocols (Bueno et al., 2014). With 120 or so hand transplants documented by the International Registry on Hand and composite tissue transplantation (Petruzzo et al., 2010), rehabilitation programs have been described across the globe, including India (Iyer et al., 2017), United States (Bueno et al., 2014), Australia, Poland and the United Kingdom (United Kingdom).

The Brigham and Women’s Hospital (BWH) protocol has four sequential phases: 1)Pre-operative to establish functional baseline and expectations; 2) Initial post-operative focusing on healing; 3) Intermediate (weeks 2–8) focusing on range of motion and strengthening; and 4) Late focusing on increasing activity and participation (Bueno et al., 2014). The United Kingdom program (Burdon et al., 2020) uses pre-habilitation as part of preoperative planning including exercises and motor imagery. Subsequent stages are early (0–6 weeks), intermediate (6–12 weeks) and late (12 weeks+), with goals of each stage similar to the corresponding latter BWH phases. Functional outcome assessment categories described include objective motor and sensory functional tests, subjective provider and patient feedback, and treatment costs (Ninkovic et al., 2011). The reported immediate clinical and functional outcomes of hand transplantation are encouraging, long-term outcomes data is only available for small samples (Kaufman and Breidenbach, 2011). Long-term clinical, activity and participation outcomes data for larger cohorts is in the process of being collected and published.

Additional considerations include requirements for Institutional Review Board (IRB) as well as institutional financial and regulatory support to move forward with the procedure. The importance of donor selection and receipt appropriateness cannot be overstated (Ravindra and Gorantla., 2011). To summarize, a detailed pre and postsurgical and community-based rehabilitation protocol is strongly recommended. Hence, in addition to clinical expertise, robust processes and organizational alignment are needed to support clinical, functional, and fiscal viability of this program. From a generalizability perspective, the value of a registry in collating process and outcomes data to facilitate global evidence-based guidelines development for this pioneering field cannot be overstated.

## Future Directions

In the near term, hand transplants will likely remain a relatively rare surgical procedure. The complexity of the surgical technique, while non-trivial, is ultimately manageable and within the technical capabilities of an experienced hand and microsurgical team given the shared experience with replantation. Similarly, immune suppression regimens have been developed, with reasonable success at minimizing rejection despite the challenges posed by transplantation of skin. Robust rehabilitation protocols at select centers further support the translation of this technical endeavor into a functional limb that can improve quality of life. However, neither technical prowess nor immune suppression are the rate limiting steps in adoption of this technology. Rather, it is the ethical underpinnings of the endeavor.

As discussed above, seamless use of the hand influences quality of life for individuals, but unlike other organ transplants, there is no evidence that hand transplants prolong lifespan, and may even shorten it. This paradox strikes at the heart of the physician’s imperative “To do no harm”. Almost all surgical indications are a balance of risks with benefits, with patient inclusion in surgical decision-making being vital, and hand transplants are no exception. The current decision process in hand transplantation is complicated by paucity of clinical data on both short- and long-term outcomes. Slow adoption by the surgical community has additionally led to a small number of hand transplants worldwide, making it difficult to accurately understand patient selection for optimal outcomes. This Catch 22 of limited evidence-limited outcomes data has continued to limit access and evidence. More information is needed about long term outcomes and utility of hand transplants, particularly when compared with upper extremity prosthesis use.

Similarly, the risk-benefit ratio can be tipped by advances in immunomodulation. As new discoveries and innovative techniques change the post-transplantation risk of lifelong immune-suppression, patients and surgeons may be more willing to proceed with non-conventional transplants. This in turn could lead to higher clinical volume and better powered studies on best-standard rehabilitation protocols and long-term outcomes.

The decision is further confounded by stunning advancements in prosthesis technology and rehabilitation techniques over the past two decades. Surgical advances such as targeted muscle reinnervation and osseointegration will continue to enhance prosthetic function, with much lower risk to patients than hand transplantation, while standardized rehabilitation protocols continue to help individuals establish focused goals and track progress over their lifetime. Still, until sensory input from these devices is addressed, patients will lack the ability to use the prosthetic device without direct visualization. However, this gap is closing as well, with new research efforts demonstrating effective sensory feedback integration into prosthetic devices (Ortiz-Catalan et al., 2020)^3^.

Going forward, we expect that future research endeavors will continue along parallel tracks in a number of areas. Continued observation of the cohort of current transplant recipients will provide insight for improved long term medical management. Additionally, ongoing work in cortical mapping and reorganization following limb loss, transplantation and prosthetic adoption will be key to understanding the potential for seamless incorporation of these technologies. Hopefully, current work on limb regeneration may someday render these techniques redundant, but until then, much can be learned about how to optimize return of function in patients suffering limb loss.

## Data Availability

The original contributions presented in the study are included in the article/Supplementary Material, further inquiries can be directed to the corresponding author.
